# The International Medical Annual

**Published:** 1902-05

**Authors:** 


THE INTERNATIONAL MEDICAL ANNUAL. A Year-Book of Treatment and Practitioners' Index. Thirty-six contributors, American and Foreign. Twentieth year. Octavo, pp. 688. New York and Chicago: E. B. Treat & Co. 1902. [Price, $3.00.]

For twenty years Treat's Annual has been before the profession as a help and guide in their daily work, and in that time it has grown from a small book of 300 pages to a large handsome volume of nearly 1,000 pages. Not alone in size has it grown, but in quality, and in its ability to keep apace with the newest thought and experience has Treat's annual succeeded and proven its worth. Today "The Annual" is a household name wherever the profession of medicine is recognised.

The 1902 Annual is built on the same broad lines as the preceding volumes—with its review of therapeutic agents and dictionary of materia medica, followed by an alphabetic review of the latest work done during the year in medicine and surgery. Special articles on such subjects as arsenical poisoning, aseptic surgery in New York, clinical examination of the blood, diseases of the ear, colony treatment of epilepsy, diseases of metabolism, formic aldehyde in the treatment of phthisis, high frequency currents in phthisis, lateral curvature of the spine, vision and *x*-rays are included in the body of the work. The annual review of sanitary science and new books add to the general usefulness of the book.

The illustrations are many and excellent and the make-up of the book is uniform with other editions. W. C. K.

